# Cognitive reserve over life course and 7-year trajectories of cognitive decline: results from China health and retirement longitudinal study

**DOI:** 10.1186/s12889-022-12671-6

**Published:** 2022-02-04

**Authors:** Xuanji Chen, Baowen Xue, Yaoyue Hu

**Affiliations:** 1 School of Public Health and Management, Chongqing Medical University, Chongqing, 400016 P. R. China; 2 Research Department of Epidemiology and Public Health, University College London, 1-19 Torrington Place, London, WC1E 6BT UK

**Keywords:** Cognitive reserve, Life course, Urban-rural context, Trajectories of cognitive decline, Aging

## Abstract

**Background:**

Cognitive reserve (CR) could partly explain the individual heterogeneity in cognitive decline. No study measured CR from a life course perspective and investigated the association between CR and trajectories of cognitive decline in older Chinese adults.

**Methods:**

Data of 6795 Chinese adults aged 60+ from China Health and Retirement Longitudinal Study were used. Global cognition score (0–32) was assessed in all four waves. A life-course CR score was constructed using markers of childhood circumstance, education, highest occupational class, and leisure activities in later life. Latent growth curve modelling (LGCM) was applied to assess the association between CR and trajectories of cognitive decline.

**Results:**

For the life-course CR, factor loadings of markers in adulthood and later life were larger than that of markers in childhood. The life-course CR score (ranged between − 2.727 and 6.537, SD: 1.74) was higher in urban Chinese adults (0.75, SD: 1.90) than in rural Chinese adults (− 0.50, SD: 1.43). The unconditional LGCM results showed that urban older Chinese adults had better global cognition at baseline (intercept: 15.010, 95% CI: 14.783, 15.237) and a slower rate of cognitive decline per year (linear slope: -0.394, 95% CI: − 0.508, − 0.281) than their rural counterparts (intercept: 12.144, 95% CI: 11.960, 12.329; linear slope: -0.498, 95% CI: − 0.588, − 0.408). After controlling for all covariates, one-unit higher CR score was associated with 1.615 (95% CI: 1.521, 1.709) and 1.768 (95% CI: 1.659, 1.876) unit higher global cognition at baseline for urban and rural older Chinese adults, respectively. The slower rate of cognitive decline associated with higher CR was more evident in rural residents (slope: 0.083, 95% CI: 0.057, 0.108) than in their urban counterparts (0.054, 95% CI: 0.031, 0.077).

**Conclusions:**

CR was associated with better baseline cognition and slower cognitive decline in Chinese older adults. Although rural residents were disadvantaged in both CR and cognition, the protective effect of CR against cognitive decline was stronger for them than in those who live in urban area.

**Supplementary Information:**

The online version contains supplementary material available at 10.1186/s12889-022-12671-6.

## Background

Population aging is a global phenomenon, and China is not an exception. In 2010, China had 168 million adults aged 60 years and older; this number is projected to reach 402 million in 2040 [1]. Along with the rapid population aging, the global burden of neurodegenerative diseases, including dementia, brain atrophy, and Parkinson’s disease, has also been drastically increasing [2]. In 2015, approximately 47 million people in the world were living with dementia, and 25% of them were from China [3]. With no cure available, preventing dementia is particularly crucial.

Stern [4] proposed the concept of cognitive reserve (CR) – the functional and dynamic abilities of the brain that actively buffer neurodegeneration and neuropathology [5] – to explain the observed heterogeneity between individuals in their cognitive decline trajectories and their susceptibility to cognitive impairments. More specifically, CR is defined as the residual variance in cognitive performance between individuals that is not explained by brain measures (e.g., brain size or neuronal count), and is not manifested by any directly measured cognitive or brain performance [6]. One example would be that, among two individuals with same level of structural brain capacity (e.g., brain size or neuronal count), the one with a higher level of CR may tolerate greater brain lesion and thus maintain better cognition than the other one [4]. In the reserve theory, CR copes with brain damage via: 1) pre-existing cognitive processes (i.e., brain networks or cognitive paradigms that are less susceptible to brain damage), and 2) enlisting compensatory process (i.e., the part of brain networks that are not engaged when the brain is not damaged) based on individual’s innate intelligence and life experience [5].

CR is often conceptualized as an unobserved multi-factorial construct shaped by indicators such as education, occupation, and leisure activities which are hypothesized to offer “protection” against neuropathology and thus maintain clinical cognitive performance [7]. According to the CR hypothesis, education is considered as a key cognitive stimulation during brain maturation in childhood and early adulthood that enhances CR[4]. Furthermore, cognitively engaged lifestyle during adulthood, such as cognitively demanding occupation, can yield additional neuronal resources that increase the level of CR, thus building up a buffer against cognitive decline at old age [4]. High-class occupations usually involve higher levels of skill discretion (i.e., variety of work and opportunities for use of skills) that requires more mental processing than low-class occupations, which also ameliorates CR [8]. For older adults, leisure time constitutes a large part of their daily life after retirement. Leisure activities in later life could help older adults to maintain or improve their CR through 1) repetition of specific skills (e.g. working memory and perceptual speed) which make them less vulnerable to underlying neuropathological changes; and 2) training of strategic cognitive skills (e.g., working memory, information processing speed) that serve as a compensatory mechanism in the CR theory [9, 10]. Previous studies nevertheless have reported mixed results on the trajectories of cognitive decline for older adults with different levels of CR measured by varied indicators [11, 12]. For example, using education to reflect CR, a systematic review found no association between CR and the rate of cognitive decline; whereas CR captured by occupation and leisure activities has often been found to be associated with cognitive decline [7, 9].

According to the life-course theory, CR is malleable throughout life [13, 14]. CR develops and cumulates through one’s life, and experiences earlier in life are imperative for cognitive function and cognitive decline at older age. One marker of CR in a specific stage of life alone could not fully capture the accumulation of CR over life time, which often leads to bias from non-reserve pathways that are relevant to other CR markers [13]. It is therefore important to capture CR across the childhood, adulthood, and later life, and to consider CR as a developmental and augmentable process that could shape the initial cognition and cognitive decline [15].

In addition to the commonly studied modifiable determinants of CR (e.g., education, occupational complexity, and leisure activities in later life) [16], life-course research of CR concerns socioeconomic status (SES) across life stages. Childhood SES (e.g., parents’ education and occupation) has been advocated to be included in capturing CR as most reserve accumulates during childhood and in young adulthood and found to be associated with cognitive function in later life [14, 17]. Studies have demonstrated the critical period for the development of cognition in early life – individuals who experienced adverse events during the critical period have lower cognition than their counterparts without such adversity [18, 19]. Higher childhood SES has been found to have a positive effect on cognitive function, although inconsistent findings were reported on the rate of cognitive decline [20, 21]. In adulthood, SES (e.g., own education and occupation) represents one’s experience accumulated through most part of the lifetime and plays an important role in successful aging [22]. Previous research has used both childhood and adulthood SES as CR markers with the rationale that higher level of SES provides an individual with better economic resources and environmental enrichment, which contributes to better brain health in both childhood and adulthood [10]. Lower SES, nevertheless, is associated with higher risk of toxic exposures and poorer health due to substandard prenatal care or malnutrition in critical period, all of which may compromise brain health [10].

In China, there is a prominent urban-rural disparity in cognition and dementia [23, 24]. Both the prevalence and incidence of dementia are lower in Chinese adults aged 55 and older who live in urban areas (prevalence: 4.4%, incidence: 6.7 per 1000 person-years) than in their rural counterparts (prevalence: 6.1%, incidence: 8.7 per 1000 person-years) [24, 25]. Since urban residents in China enjoy more favorable material, working, and social conditions through life time compare to rural residents, they may have higher levels of CR that explains the urban-rural disparity in cognition and dementia. However, the majority of previous studies from China are cross-sectional, and the longitudinal association between CR and trajectories of cognitive decline in the urban-rural setting in China has not been examined. This study applies a life-course approach to capture CR by different markers across childhood, adulthood and later life, and to assess how life-course CR was associated with later life cognitive decline trajectories over 7 years in Chinese urban and rural residents aged 60 and over.

## Methods

### Study design

Data came from the China Health and Retirement Longitudinal Study (CHARLS) – a nationally representative cohort study of community-dwelling Chinese population aged 45 and older. The baseline survey (Wave 1) was conducted in 2011 with 17,708 participants from 150 counties and districts of 28 provinces. Comprehensive information on sociodemographic characteristics, family structure, health status and functioning, health behaviors, work and retirement were collected via face-to-face computer-assisted interview. Ethical approval was provided by the Ethical Review Committee of Peking University. Three follow-up surveys were carried out in 2013 (Wave 2), 2015 (Wave 3), and 2018 (Wave 4). Details of CHARLS are provided elsewhere [26]. We included CHARLS participants aged 60 and older at baseline who had at least one assessment of cognition in Waves 1–4 (*N* = 6795, Supplementary Fig. 1).

### Cognition

Global cognition with three components – memory, executive function, and time orientation [27] – was assessed in all four waves. Memory was measured by immediate and delayed recall of ten words. A memory score (0–20) was calculated by summing up the number of correctly recalled words. Time orientation (0–4) was assessed by naming the day of the week, the month, the date of the month, and the year, with one score given for each correct answer. The executive function tests consisted of serial 7 subtractions from 100 (up to five times, with one score given for each correct answer) and drawing of overlapped pentagons (three scores were given for a successful drawing). The executive score was the sum of these two tests (0–8). The sum of all three scores reflected the participants’ global cognitive function (0–32) [28].

### Life-course cognitive reserve

We used childhood circumstances, highest educational attainment, highest occupational class, and leisure activities in later life to capture participants’ CR. These indicators do not constitute CR but contribute to CR or proxies of CR. Childhood circumstances covered childhood financial situation, highest level of parents’ education and occupation, starvation, and participants’ height. Height was included because, apart from genetic heritability, it is highly influenced by the nutrition status in childhood; and it was found to be associated with cognition in later life [29]. All the CR markers were measured at baseline except childhood financial situation, parents’ education and occupation, and starvation which were collected at the Life Event History Wave in 2014. Measurements of CR indicators are summarized in Supplementary Table 1.

### Covariates

All covariates were measured at baseline. Urban-rural residence was based on the categorization defined by Chinese National Bureau of Statistics. Besides sociodemographic factors (age, gender, and marital status), we also included health behaviors, doctor-diagnosed chronic diseases, self-reported hearing loss, probable depression, and total number of difficulties in activities of daily living (ADLs) at baseline as covariates [30]. Hearing loss was included as a covariate because it was reported to be associated with impaired performance across cognitive domains and increased risk of dementia, and could contribute to the development of social isolation, loneliness, and reduced leisure activities in later life [31]. Health behaviors consisted of body mass index (BMI), smoking, and frequency of alcohol drinking. BMI was categorized into underweight (< 18.5), normal (18.5–23.9), overweight (24.0–27.9) and obese (≥28.0) using the cut-off points recommended for the Chinese population [32]. Smoking was grouped into never, former or current smoking. The participants reported their frequency of drinking beer, wine, and liquor in the past year (never, < 1/month, 1–3/month, 1–6/week, ≥1/day). Doctor-diagnosed chronic diseases contained hypertension, dyslipidemia, diabetes, heart diseases, and stroke. Probable depression was assessed by the ten-item Center for Epidemiologic Studies Depression Scale (CESD-10) [33] and determined using a cut-off of 12 [34]. ADLs covered dressing, bathing, eating, getting out of bed, toileting, and continence.

### Statistical analysis

Since the life-course CR is theorized at a higher level built upon CR in childhood, adulthood, and later life, a second-order confirmatory factor analysis (CFA) was applied to construct a CR score [35]. Three first-order latent factors were generated to capture: 1) CR in childhood using markers of childhood circumstances; 2) CR in adulthood using highest educational attainment and highest occupational class; and 3) CR in later life reflected by leisure activities. Then, a second-order factor of life-course CR was estimated based on the first-order factors.

Latent growth curve modelling (LGCM) was used to estimate the trajectories of cognitive decline over the 7 years of follow-up. LGCM is flexible and powerful for understanding individual changes over time with multi-wave data available [36]. First, we fitted an unconditional LGCM with three latent factors – intercept, slope, and quadratic slope – to determine the shape of the trajectories of cognitive decline. The intercept factor reflects the initial level of cognition at baseline, whereas the slope factor captures the linear rate of decline in cognition and the quadratic slope specifies the possible non-linear cognitive decline [36]. For the slope and quadratic slope, factor loadings of 0, 2, 4, 7 were used to reflect the time intervals between waves. The following indices were used to assess the goodness of model fit: comparative fit index (CFI) > 0.95, Tucker-Lewis index (TLI) > 0.95, and root mean square error of approximation (RMSEA) ≤0.05 [37, 38]. Our model showed good model fit (CFI = 0.991, TLI = 0.987, RMSEA = 0.049).

Since we found an interaction between CR and urban-rural residence on the slope (*p* = 0.044) but not on the intercept (*p* = 0.129), our sample was stratified by the urban-rural residence. Both the intercept and slope were controlled for gender, age, and age squared (Model 1), additionally for marital status, health behaviors (Model 2) and for doctor-diagnosed chronic diseases, hearing loss, probable depression, and ADLs (Model 3). The quadratic slope was not regressed on CR or any covariates because it was too small and lacks heterogeneity between individuals (0.012 in urban residents, 0.014 in rural residents). Missing data was handled by full information maximum likelihood estimation (FIML) which is valid under missing at random assumption (i.e. missingness depends on observed variables) and is as good as multiple imputation [39–42]. As using a mixed sample of cognitively intact individuals and those with mild cognitive impairment (MCI) may introduce bias [12], sensitivity analysis was carried out by excluding participants with MCI at baseline (i.e. whose global cognition score was lower than one standard deviation below the mean for their age- and education-matched peers) [43]. Data analyses were performed using R version 3.6.3 (R Core Team, 2020) and Mplus version 8.3 (Muthén & Muthén, 2019). Ageing vector graph was presented to show the global cognition at baseline, the direction and rate of cognitive decline by CR level for every 2 years of age over the follow-up.

All methods were performed in accordance with the Declaration of Helsinki.

## Results

Table 1 presents the sample characteristics (men = 3459, women = 3336). Distribution of the CR markers are summarized in Supplementary Table 2. Over 40% of the participants reported being starved at age 6–12 and 13–17 (Supplementary Table 2). The majority of the participants’ parents did not receive formal education and were agricultural workers. Most of the participants received elementary school and lower education and worked in agricultural jobs. Visiting friends was the most common leisure activity in later life. Compared to men, women tended to be older, live in urban area, have less education, have agricultural related occupations, have lower CR scores, and have lower level of cognition at baseline.

**Table 1:** Sample characteristics at baseline (*N* = 6795)

	Men	Women
	(***N*** = 3459)	(***N*** = 3336)
**Baseline cognition** (mean, SD)	14.78 (5.49)	12.07 (6.00)
**Cognitive reserve** (mean, SD)	0.53 (1.62)	−0.55 (1.62)
**Mean age** (years, SD)	67.67 (6.29)	67.78 (6.61)
**Urban-rural residence** (%)		
Urban	1330 (38.5)	1371 (41.1)
Rural	2129 (61.5)	1965 (58.9)
**Marital Status** (%)		
Married	3020 (87.3)	2382 (71.4)
Unmarried	439 (12.7)	953 (28.6)
Missing	0 (0.0)	1 (0.0)
**Alcohol drinking** (%)		
Never	2076 (60.0)	3090 (92.6)
< 1/month	44 (1.3)	14 (0.4)
1–3/month	95 (2.7)	29 (0.9)
1–6/week	212 (6.1)	36 (1.1)
≥1/day	690 (19.9)	60 (1.8)
Missing	342 (9.9)	107 (3.2)
**Smoking status** (%)		
Never	935 (27.0)	2949 (88.4)
Former	686 (19.8)	102 (3.1)
Current	1728 (50.0)	259 (7.8)
Missing	110 (3.2)	26 (0.8)
**BMI** (%)		
Underweight	290 (8.4)	275 (8.2)
Normal	1719 (49.7)	1327 (39.8)
Overweight	631 (18.2)	814 (24.4)
Obese	192 (5.6)	342 (10.3)
Missing	627 (18.1)	578 (17.3)
**Hypertension** (%)		
No	2348 (67.9)	2053 (61.5)
Yes	1088 (31.5)	1252 (37.5)
Missing	23 (0.7)	31 (0.9)
**Diabetes** (%)		
No	3195 (92.4)	2993 (89.7)
Yes	235 (6.8)	300 (9.0)
Missing	29 (0.8)	43 (1.3)
**Heart disease** (%)		
No	2895 (83.7)	2672 (80.1)
Yes	531 (15.4)	626 (18.8)
Missing	33 (1.0)	38 (1.1)
**Stroke** (%)		
No	3290 (95.1)	3193 (95.7)
Yes	147 (4.2)	117 (3.5)
Missing	22 (0.6)	26 (0.8)
**Dyslipidemia** (%)		
No	3055 (88.3)	2864 (85.9)
Yes	335 (9.7)	396 (11.9)
Missing	69 (2.0)	76 (2.3)
**Hearing loss** (%)		
No	2972 (85.9)	2917 (87.4)
Yes	474 (13.7)	400 (12.0)
Missing	13 (0.4)	19 (0.6)
**Probable depression**^**a**^ (%)		
No	2410 (69.7)	1863 (55.8)
Yes	820 (23.7)	1235 (37.0)
Missing	229 (6.6)	238 (7.1)
**ADLs** (%)		
0	2766 (80.0)	2434 (73.0)
1	355 (10.3)	409 (12.3)
2	132 (3.8)	204 (6.1)
3	81 (2.3)	124 (3.7)
4	58 (1.7)	74 (2.2)
5	36 (1.0)	53 (1.6)
6	20 (0.6)	24 (0.7)
Missing	11 (0.3)	14 (0.4)

*Notes*: *ADLs* activities of daily life, *BMI* body mass index, *SD* standard deviation

^a^ Probable depression was defined as having CES-D-10 score ≥ 12

**Fig. 1:**
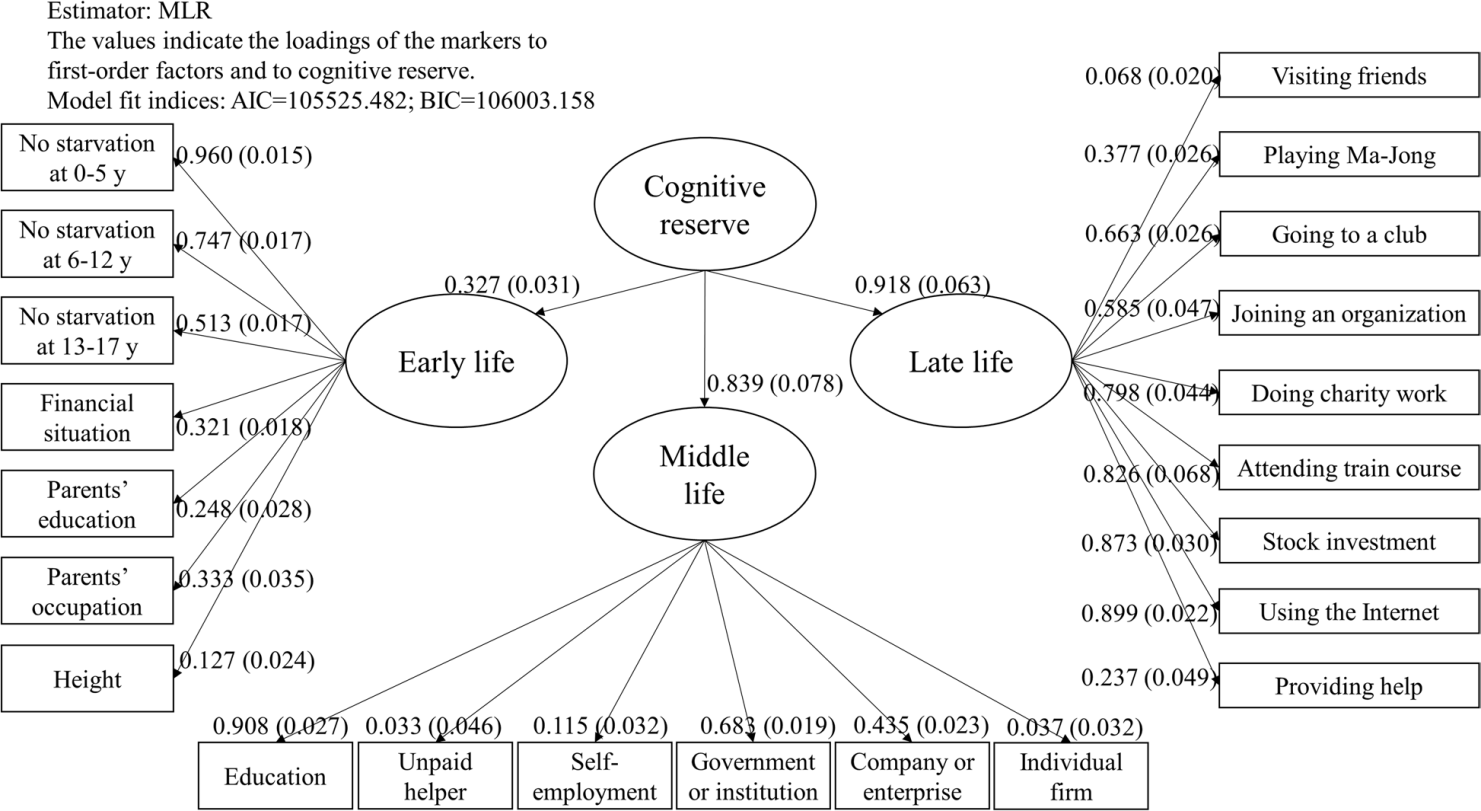
Second-order confirmatory factor analysis for life-course cognitive reserve

The standardized second-order CFA results are shown in Fig. 1. The factor loadings of CR proxies from adulthood (adulthood SES, 0.839, standard error [SE]: 0.078) and from later life (later life activities, 0.918, SE: 0.063) were larger than that of CR proxies from childhood (childhood SES, 0.327, SE: 0.031). Within each first-order factor, childhood starvation, education, being employed by government/institution, using the Internet, doing stock investment, attending training course, and doing charity work were more related to CR scores in childhood (childhood SES), adulthood (adulthood SES), later life (leisure activities), respectively. The life-course CR score generated from the CFA ranged between − 2.727 and 6.537 (standard deviation [SD]: 1.74), with the higher value indicating higher level of CR accumulated over life time. The CR score was higher in urban older Chinese adults (mean: 0.75, SD: 1.90) than in their rural counterparts (− 0.50, SD: 1.43).

The unconditional LGCM results showed that the urban older Chinese adults had better global cognition at baseline (Supplementary Table 3, intercept: 15.010, 95% CI: 14.783, 15.237) and a slower rate of cognitive decline per year (linear slope: -0.394, 95% CI: − 0.508, − 0.281; quadratic slope: 0.012, 95% CI: − 0.005, 0.029) than in rural older Chinese (intercept: 12.144, 95% CI: 11.960–12.329; linear slope: -0.498, 95% CI: − 0.588, − 0.408; quadratic slope: 0.014, 95% CI: 0.001, 0.027). The quadratic slope was not regressed by CR score or covariates because it was too small thus lacks individual heterogeneity. The LGCM results on the trajectories of cognitive decline by CR score in urban and rural older Chinese adults are shown in Tables 2 and 3, respectively. In Model 1, one-unit higher CR score was associated with 1.742 (95% CI: 1.652, 1.833) and 1.852 (95% CI: 1.743, 1.961) unit higher global cognition at baseline (intercept) for urban and rural residents, respectively. Although cognition decreased faster in rural residents than in urban residents, the slower rate of cognitive decline associated with higher CR score was more evident in rural residents (slope: 0.083, 95% CI: 0.058, 0.108) than in their urban counterparts (0.048, 95% CI: 0.026, 0.071). Controlling for marital status and health behaviors in Model 2 did not change the results. Adjustments of chronic diseases, hearing loss, probable depression and ADLs in Model 3 weakened the association between CR score and the intercept in both urban and rural residents, but it strengthened the relationship between CR score and the rate of cognitive decline over time in urban residents (0.055, 95% CI: 0.032, 0.079) while the slope remained unchanged for their rural counterparts.

**Table 2:** Seven-year trajectories of global cognition (0–32) by cognitive reserve in urban residents (*N* = 2701)

	Model 1^a^		Model 2^b^		Model 3^c^	
	Intercept	Slope	Intercept	Slope	Intercept	Slope
	b (95% CI)	b (95% CI)	b (95% CI)	b (95% CI)	b (95% CI)	b (95% CI)
**Mean**	15.362 (14.972, 15.751)	−0.293 (− 0.429, − 0.156)	14.562 (13.858, 15.266)	− 0.233 (− 0.432, − 0.035)	15.140 (14.429, 15.851)	− 0.287 (− 0.489, − 0.085)
**Variance**	8.462 (7.211, 9.714)	0.136 (0.062, 0.211)	8.269 (7.026, 9.512)	0.131 (0.057, 0.205)	7.912 (6.697, 9.127)	0.131 (0.057, 0.205)
**Cognitive reserve**	1.742 (1.652, 1.833)^*^	0.048 (0.026, 0.071)^*^	1.714 (1.623, 1.805)^*^	0.047 (0.024, 0.069)^*^	1.615 (1.521, 1.709)^*^	0.054 (0.031, 0.077)^*^
**Baseline age, centred**	−0.157 (− 0.228, − 0.086)^*^	−0.021 (− 0.040, − 0.002)^*^	−0.146 (− 0.217, − 0.074)^*^	−0.020 (− 0.039, 0.000)	−0.148 (− 0.219, − 0.078)^*^	−0.019 (− 0.038, 0.000)
**Baseline age, squared**	− 0.002 (− 0.005, 0.002)	0.000 (− 0.001, 0.001)	−0.001 (− 0.005, 0.002)	0.000 (− 0.001, 0.001)	− 0.001 (− 0.004, 0.002)	0.000 (− 0.001, 0.001)
**Gender**						
Men	Ref	–	–	–	–	–
Women	−0.543 (− 0.881, − 0.204)^*^	0.092 (0.011, 0.172)^*^	−0.455 (− 0.898, − 0.013)^*^	0.021 (− 0.086, 0.127)	−0.364 (− 0.804, 0.077)	0.003 (− 0.103, 0.110)
**Marital status**						
Unmarried			Ref	–	–	–
Married			0.352 (−0.092, 0.797)	0.039 (−0.074, 0.151)	0.272 (− 0.167, 0.711)	0.042 (− 0.071, 0.154)
**Alcohol drinking**						
Never			Ref	–	–	–
1/month			1.373 (−0.589, 3.334)	−0.199 (− 0.686, 0.289)	1.125 (− 0.809, 3.060)	−0.177 (− 0.664, 0.310)
2–3/month			0.541 (−0.728, 1.810)	0.051 (− 0.250, 0.353)	0.415 (− 0.837, 1.667)	0.075 (− 0.227, 0.376)
1–6/week			0.112 (− 0.827, 1.051)	−0.163 (− 0.390, 0.064)	0.126 (− 0.801, 1.052)	−0.176 (− 0.403, 0.051)
≥1/day			0.078 (−0.507, 0.663)	0.027 (−0.110, 0.164)	0.027 (− 0.551, 0.606)	0.029 (− 0.108, 0.166)
**Smoking status**						
Never			Ref	–	–	–
Former			0.427 (−0.124, 0.979)	−0.141 (− 0.276, − 0.007)^*^	0.505 (− 0.040, 1.050)	−0.150 (− 0.284, − 0.015)^*^
Current			− 0.069 (− 0.528, 0.391)	− 0.122 (− 0.231, − 0.012)^*^	−0.035 (− 0.489, 0.418)	−0.123 (− 0.232, − 0.014)^*^
**BMI**						
Underweight			−0.324 (−1.122, 0.474)	0.034 (− 0.169, 0.238)	−0.266 (− 1.059, 0.526)	0.031 (− 0.173, 0.235)
Normal			Ref	–	–	–
Overweight			0.791 (0.370, 1.212)^*^	0.017 (−0.082, 0.116)	0.738 (0.314, 1.161)^*^	0.018 (− 0.082, 0.119)
Obesity			0.553 (−0.007, 1.112)	− 0.057 (− 0.188, 0.075)	0.457 (− 0.116, 1.030)	− 0.059 (− 0.196, 0.077)
**Hearing loss**						
No					Ref	–
Yes					−0.428 (− 0.975, 0.119)	−0.011 (− 0.155, 0.133)
**Hypertension**						
No					Ref	–
Yes					−0.183 (− 0.544, 0.177)	0.006 (−0.082, 0.094)
**Diabetes**						
No					Ref	–
Yes					0.035 (−0.485, 0.556)	− 0.054 (− 0.181, 0.073)
**Heart diseases**						
No					Ref	–
Yes					0.194 (−0.207, 0.595)	−0.004 (− 0.102, 0.095)
**Stroke**						
No					Ref	–
Yes					−0.243 (−1.084, 0.599)	− 0.191 (− 0.428, 0.047)
**Dyslipidaemia**						
No					Ref	–
Yes					0.166 (−0.304, 0.637)	0.088 (− 0.026, 0.202)
**Probable depression**^§^						
No					Ref	–
Yes					−1.059 (−1.468, − 0.649)^*^	0.094 (− 0.009, 0.197)
**ADLs (0–6)**					−0.487 (− 0.668, − 0.306)^*^	0.064 (0.012, 0.117)^*^

*Notes*: *ADLs* activities of daily life, *BMI* body mass index, *Ref* reference category. Quadratic slope was not regressed on CR or covariates because the magnitude of the slope is too small (b = 0.012). ^a^ Adjusted for age, age squared, gender, and urban/rural residence. ^b^ Adjusted for Model 1 covariates plus smoking status, alcohol drinking frequency and BMI. ^c^ Adjusted for Model 2 covariates plus number of limitations in ADLs, self-rated hearing, probable depression, and self-reported doctor-diagnosis of cardiovascular disease, hypertension, diabetes, stroke and dyslipidaemia. §Probable depression was defined as having CES-D-10 score ≥ 12. ^*^ *p* < 0.05

**Table 3:** Seven-year trajectories of global cognition (0–32) by cognitive reserve in rural residents (*N* = 4094)

	Model 1^a^		Model 2^b^		Model 3^c^	
	Intercept	Slope	Intercept	Slope	Intercept	Slope
	b (95% CI)	b (95% CI)	b (95% CI)	b (95% CI)	b (95% CI)	b (95% CI)
**Mean**	15.150 (14.834, 15.466)	−0.337 (− 0.488, − 0.226)	14.989 (14.420, 15.557)	− 0.343 (− 0.499, − 0.186)	15.489 (14.915, 16.062)	− 0.386 (− 0.545, − 0.227)
**Variance**	8.628 (7.562, 9.695)	0.074 (0.014, 0.133)	8.508 (7.444, 9.571)	0.070 (0.011, 0.129)	7.844 (6.808, 8.880)	0.064 (0.005, 0.123)
**Cognitive reserve**	1.852 (1.743, 1.961)^*^	0.083 (0.058, 0.108)^*^	1.839 (1.730, 1.948)^*^	0.080 (0.055, 0.105)^*^	1.768 (1.659, 1.876)^*^	0.083 (0.057, 0.108)^*^
**Baseline age, centred**	−0.229 (− 0.292, − 0.167)^*^	−0.024 (− 0.040, − 0.008)^*^	−0.223 (− 0.286, − 0.159)^*^	−0.023 (− 0.039, − 0.008)^*^	−0.207 (− 0.270, − 0.145)^*^	−0.025 (− 0.040, − 0.009)^*^
**Baseline age, squared**	0.002 (− 0.001, 0.005)	0.000 (0.000, 0.001)	0.002 (− 0.001, 0.005)	0.000 (0.000, 0.001)	0.002 (− 0.001, 0.004)	0.000 (0.000, 0.001)
**Gender**						
Men	Ref	–	–	–	–	–
Women	−1.111 (− 1.415, − 0.806)^*^	0.023 (− 0.047, 0.093)	− 1.155 (− 1.552, − 0.757)^*^	−0.025 (− 0.115, 0.065)	−1.015 (− 1.408, − 0.622)^*^	−0.035 (− 0.126, 0.055)
**Marital status**						
Unmarried			Ref	–	–	–
Married			0.134 (−0.229, 0.498)	0.067 (−0.020, 0.155)	0.048 (− 0.311, 0.406)	0.072 (− 0.016, 0.159)
**Alcohol drinking**						
Never			Ref	–	–	–
1/month			−0.804 (−2.184, 0.576)	0.288 (−0.025, 0.602)	− 0.560 (−1.920, 0.800)	0.287 (− 0.026, 0.601)
2–3/month			− 0.393 (− 1.374, 0.587)	0.012 (− 0.201, 0.225)	−0.309 (− 1.273, 0.654)	−0.005 (− 0.217, 0.208)
1–6/week			0.128 (−0.579, 0.834)	0.014 (− 0.141, 0.169)	0.083 (− 0.612, 0.778)	0.021 (− 0.134, 0.176)
≥1/day			−0.244 (− 0.693, 0.206)	0.014 (− 0.087, 0.115)	−0.350 (− 0.792, 0.093)	0.029 (− 0.071, 0.130)
**Smoking status**						
Never			Ref	–	–	–
Former			0.160 (−0.351, 0.671)	−0.061 (− 0.178, 0.057)	0.386 (− 0.118, 0.890)	−0.084 (− 0.202, 0.034)
Current			0.046 (−0.345, 0.437)	−0.096 (− 0.183, − 0.009)^*^	0.087 (− 0.297, 0.471)	−0.100 (− 0.187, − 0.013)^*^
**BMI**						
Underweight			−0.515 (− 0.992, − 0.037)^*^	−0.037 (− 0.151, 0.077)	−0.399 (− 0.870, 0.072)	−0.044 (− 0.158, 0.071)
Normal			Ref	–	–	–
Overweight			0.459 (0.086, 0.832)^*^	−0.021 (− 0.105, 0.063)	0.367 (− 0.005, 0.739)	−0.017 (− 0.102, 0.069)
Obesity			−0.152 (− 0.748, 0.445)	0.102 (− 0.033, 0.238)	−0.237 (− 0.841, 0.367)	0.108 (− 0.032, 0.248)
**Hearing loss**						
No					Ref	–
Yes					−0.934 (−1.334, − 0.535)^*^	0.089 (− 0.011, 0.189)
**Hypertension**						
No					Ref	–
Yes					0.070 (−0.242, 0.382)	−0.013 (− 0.086, 0.059)
**Diabetes**						
No					Ref	–
Yes					0.318 (−0.299, 0.935)	−0.084 (− 0.231, 0.062)
**Heart diseases**						
No					Ref	–
Yes					0.300 (−0.114, 0.715)	0.067 (− 0.029, 0.164)
**Stroke**						
No					Ref	–
Yes					−0.329 (−1.065, 0.407)	−0.061 (− 0.242, 0.120)
**Dyslipidaemia**						
No					Ref	–
Yes					0.043 (−0.497, 0.583)	0.034 (− 0.086, 0.155)
**Probable depression**^§^						
No					Ref	–
Yes					−1.030 (−1.338, −0.723)^*^	0.059 (−0.013, 0.131)
**ADLs (0–6)**					−0.371 (− 0.499, − 0.242)^*^	0.037 (0.004, 0.070)^*^

*Notes*: *ADLs* activities of daily life, *BMI* body mass index, *Ref* reference category. Quadratic slope was not regressed on CR or covariates because the magnitude of the slope is too small (b = 0.014). ^a^ Adjusted for age, age squared, gender, and urban/rural residence. ^b^ Adjusted for Model 1 covariates plus smoking status, alcohol drinking frequency and BMI. ^c^ Adjusted for Model 2 covariates plus number of limitations in ADLs, self-rated hearing, probable depression, and self-reported doctor-diagnosis of cardiovascular disease, hypertension, diabetes, stroke and dyslipidaemia. §Probable depression was defined as having CES-D-10 score ≥ 12. ^*^ *p* < 0.05

**Fig. 2:**
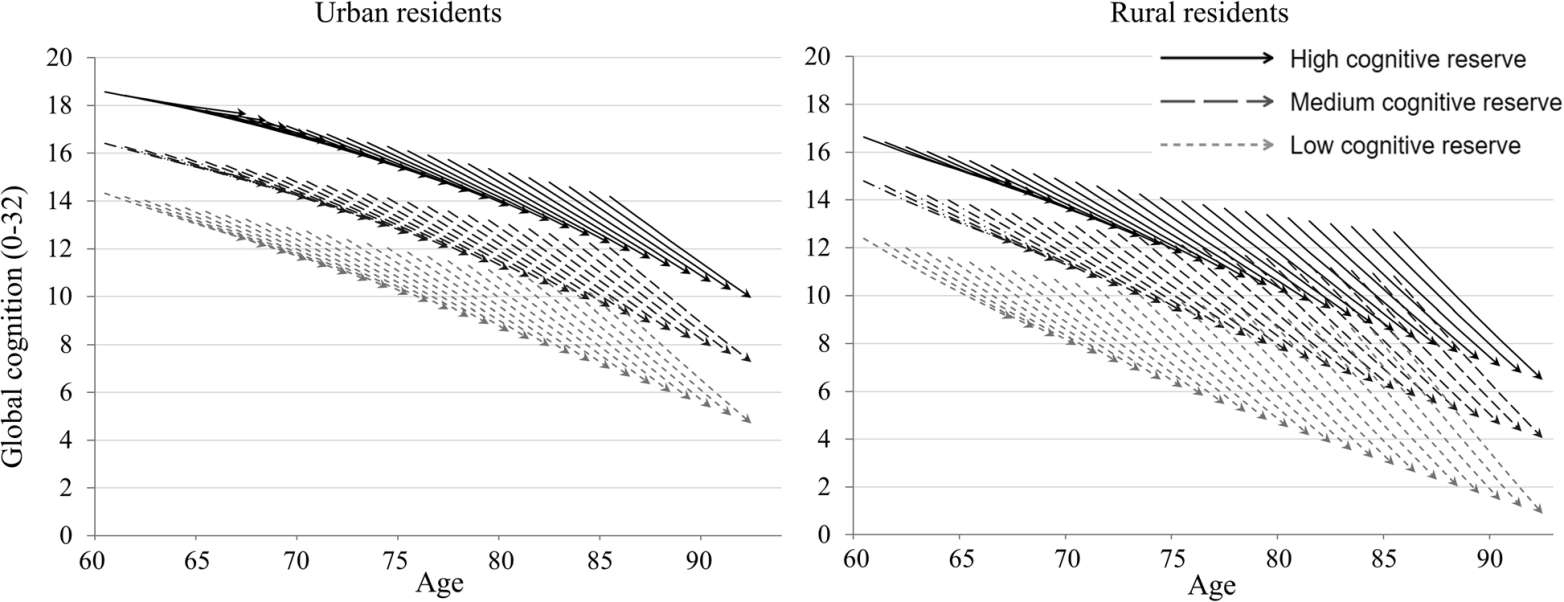
Ageing vector graph of 7-year trajectories of cognition decline by urban-rural residence and cognitive reserve levels

Figure 2 shows the trajectories of cognitive decline by tertiles of CR score in urban and rural older Chinese over the 7 years of follow-up. Despite the greater protective effect of CR in rural older Chinese adults, with smaller CR score, worse cognition at baseline and faster rate of cognitive decline, rural residents’ gaps in cognitive function between low, middle, and high CR groups widened more markedly over time than urban residents.

Sensitivity analysis of LGCM by gender and by whether participants had MCI yielded similar pattern of results (Supplementary Tables 4, 5, 6, 7).

## Discussion

In this large cohort study of 6795 middle-aged and older Chinese with 7 years of follow-up, we constructed a CR score using the life-course approach with indicators of CR from childhood, adulthood, and later life. Overall, markers in adulthood and in later life contributed more to the life-course CR score than childhood markers did. Higher life-course CR score was associated with better global cognition at baseline and a slower rate of cognitive decline over time in both urban and rural older Chinse adults, but the protective effect of CR on cognitive decline was greater in rural residents compared to their urban counterparts.

Previous studies have demonstrated positive effects of higher childhood SES on cognitive performance at baseline but provided inconsistent findings on the rate of cognitive decline [20, 21]. One study from the US measured childhood SES using parents’ education among individuals aged 24–75 and gained similar results to our study [20]. In another study of older adults aged 50+ from Europe [21], childhood SES measured by number of books in the house, crowdedness of room and house infrastructure was negatively associated with cognitive decline. By analyzing the CHARLS data, Sha et al [44] found no relationship between childhood SES and the rate of cognitive decline in Chinese older adults aged 60+. By including more comprehensive markers to reflect participants’ childhood circumstances, we found that no starvation in childhood was important for life-course CR which protects against cognitive decline at older age.

Similar to our study, by using the second-order CFA with two first-order factors (education and leisure activities) to capture CR, a recent study also showed a positive effect of CR on baseline cognitive performance and cognitive decline in older adults with subjective cognitive complaints [45]. The discrepancy between studies on the association between adulthood and/or later-life CR and cognition could be attributed, at least in part, to differences in the measurement of CR. When only considering education as a proxy of CR, studies are more likely to yield null association between CR and the rate of cognitive decline [46, 47]. This may be partly explained by the hypothesis that education contributes more to the crystallized intelligence but less to the fluid intelligence, the former of which consistently accumulates throughout life time and remains relatively stable but the latter arguably declines as early as from middle age [48]. Education may also protect against cognitive decline in later life through other pathways. For example, higher level of education is associated with higher income, the latter is also associated with slower decline in cognition via access to high-quality health care, decreased exposure to stressors, increased participation in cognitively complex occupations and/or leisure activities [49]. Another possible explanation relates to the level of education. A previous study showed that education up to 8 years was independently associated with slower cognitive decline in later life, whereas the association between more years of education (≥9) and cognitive decline was fully mediated by income [50]. Early education may promote cognitive and neural developments during the sensitive period in childhood [51].

Occupational complexity reflects the accumulation of CR over ones’ working life. Higher occupational complexity was found to be associated with better baseline cognition and slower cognitive decline after retirement [52]. In our study, higher occupational class (working in government/institution or in company/enterprise) had higher loadings to the adulthood SES than other types of work did, implying that higher occupational complexity weights more in high CR. Our results support the differential preservation theory or the mechanisms of a “use it or lose it” model [53], which hypothesizes that the rate of age-related cognitive decline is less pronounced for individuals who are more cognitively active. This hypothesis is also supported by the larger factor loadings of attending training course, doing stock investment and using the Internet for CR in later life, suggesting that these activities which heavily involve cognitive training are crucial in slowing down cognitive decline in later life.

Previous studies have shown that experiences in adulthood and later life are imperative in preventing cognitive decline at older age [15, 54]. Our second-order CFA results showed that the factor loadings of adulthood SES and leisure activities in later life on the life-course CR were larger than those of childhood SES. This finding however should be interpreted with caution as it may only reflect the close correlation between the adulthood and later-life CR indicators. Our finding also suggests that high adulthood SES and cognitively challenging activities at leisure time may buffer some of the harmful effects of adverse experiences in childhood on cognition. There are three possible mechanisms: 1) cognitive stimulation (e.g., cognitive occupational demands, leisure activities) is vital in influencing brain plasticity – a key element of CR; 2) the ability of the brain to respond to environmental stimuli by adding new neurons or by activating compensatory processes can be sustained in late life; 3) the cognitive protection provided by early-life experiences have been exhausted [15, 55].

In our study, the association between life-course CR score and the rate of cognitive decline differed between rural and urban older Chinese adults. Previous studies have reported urban-rural disparities in cognition and prevalence of dementia in China [23, 24], which may be partly explained by the differences in educational attainment between urban and rural residents. Nevertheless, in our study, even after taking education into account in measuring CR score, we still found a stronger positive effect of life-course CR score on the rate of cognitive decline in rural older Chinese adults than in their urban counterparts. One possible explanation is that CR may play a more important role in cognitive stimulation in individuals with adverse condition; whereas for individuals with relatively favorable condition, such as living in urban area, the effect of CR stimulation could be attenuated. Besides, environmental factors (e.g., neighborhood infrastructures, employment service and modern facilities) may be more critical to rural residents due to their limited resources (e.g., receiving education, having high complexity jobs, doing stock investment and using the Internet). A recent study reported a similar urban-rural discrepancy in the relationship between neighborhood environment and cognitive decline among middle-aged and older Chinese adults [56], suggesting that rural older Chinese adults may rely and benefit more from environmental factors for cognitive stimulation than their urban counterparts do. This hypothesis can be examined using longitudinal studies comparing rural residents, urban residents, rural-to-urban migrants, and urban-to-rural migrants. Alternatively, the lower baseline cognition that we observed in rural residents was probably due to their greater neuropathological lesion. According to the compensation mechanism proposed by Stern [4], individuals with neuropathological lesion may use brain structures or networks that are not normally used by individuals with intact brains in order to compensate the brain damages. The effect of compensation may be stronger in rural residents and thus explains the larger protect effect of CR on their cognitive decline.

To our knowledge, this is the first study in China which measured CR using a life-course approach and analyzed longitudinal data from a large, nationally representative sample of older Chinese adults. Combining a number of markers to capture the life-course CR makes the measurement of CR more precise and less biased by factors unrelated to CR [57]. The life-course CR enabled us to advance our understanding on the association between CR and trajectories of cognitive decline in China, and more importantly, how it differed between rural and urban older Chinese adults. The loss to follow-up was dealt with using the full information maximum likelihood which is valid under missing at random assumption and is as efficient as multiple imputation [39–42]. However, practice effect may exist in our study, which leads to under-estimated differences in cognitive decline by CR levels as the practice effect may be larger in Chinese older adults with higher CR score. Since all the markers of CR were self-reported, recall bias may occur, particularly for those occurred in childhood. Due to restrictions of the data, neuroimaging and biomarkers related to cognitive decline were not measured and thus not included in this study. Future research that further explores the mechanisms linking life-course CR, brain reserve, and genetic risk factors to the trajectories of cognitive decline is needed.

In conclusion, using a life-course approach, we found that higher life-course CR was associated with both better global cognition at baseline and slower rate of cognitive decline over time in older Chinese adults. Rural older Chinese adults had worse baseline cognition and faster cognitive decline than those living in urban areas. The protective effect of CR on the rate of cognitive decline however was more evident in rural older Chinese adults. It is beneficial to enhance CR at any stage of the life time to prevent cognitive impairments and dementia at older age. Policy makers should pay more attention to provide rural older Chinese adults with better cognitive health care due to their adverse material condition and limited resources. To reduce the rural-urban discrepancy in CR and cognitive decline in China, it is important to provide better education, create more complex jobs, and build more modernized infrastructure in rural China.

## Supplementary Information

**Additional file 1.**

## Data Availability

The CHARLS is open-access public use files and free to request online at charls.pku.edu.cn/.
