# Supplementary material for: Cognitive reserve over life course and 7-year trajectories of cognitive decline: results from China health and retirement longitudinal study
Source: BMC Public Health. 2022 Feb 4;22:231. doi: 10.1186/s12889-022-12671-6 (PMC8815141; doi:10.1186/s12889-022-12671-6)
Supplement: Supplementary file 1 — Additional file 1. [file 12889_2022_12671_MOESM1_ESM.pdf]

**Additional files**

Additional file 1:
Supplementary Figure 1. Sample inclusion and exclusion.
Supplementary Table 1. Definitions of cognitive reserve markers.
Supplementary Table 2. Sample characteristics of cognitive reserve markers.
Supplementary Table 3. Unconditional latent growth curve models.
Supplementary Table 4. Seven-year trajectories of global cognition (0-32) by cognitive reserve in men (N=3,459).
Supplementary Table 5. Seven-year trajectories of global cognition (0-32) by cognitive reserve in women (N=3,336).
Supplementary Table 6. Seven-year trajectories of global cognition (0-32) by cognitive reserve in adults with mild cognitive impairments (N=1,006).
Supplementary Table 7. Seven-year trajectories of global cognition (0-32) by cognitive reserve in adults with normal cognition (N=5,789)

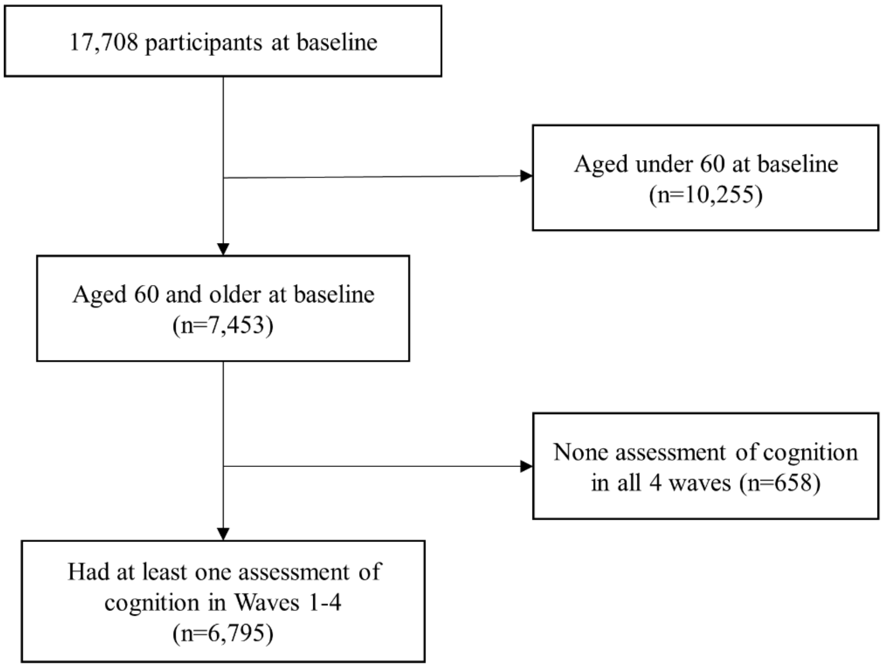


**Supplementary Figure 1. Sample inclusion and exclusion**

**Supplementary Table 1. Definitions of cognitive reserve markers**

| Cognitive reserve markers | Definitions |
|---|---|
| **Childhood** | |
| Parents' education | No formal education/illiterate=1, capable of reading and/or writing=2, sishu=3, elementary school=4, middle school=5, high school=6, vocational school=7, two/three-year college/associate degree=8, four-year College/bachelor's degree=9 |
| Parents' occupation | Agricultural=0, non-agricultural=1 |
| No starvation at 0-5 years old | Yes=1, no=0 |
| No starvation at 6-12 years old | Yes=1, no=0 |
| No starvation at 13-17 years old | Yes=1, no=0 |
| Financial situation | Worst=1, worse=2, average=3, better=4, best=5 |
| Height | Continuous |
| **Adulthood** | |
| Education | No formal education/illiterate=1, capable of reading and/or writing=2, sishu=3, elementary school=4, middle school=5, high school=6, vocational school=7, two/three-year college/associate degree=8, four-year college/bachelor's degree=9, post-graduate (master)=10 |
| Highest occupational class | 6 categories were turned into dummies: "farmer" (reference), "unpaid helper", "self-employment", "government or institution", "company/enterprise", "individual firm" |
| **Later life** | |
| Visiting friends | Never=0, seldom=1, every week=2, everyday=3 |
| Playing Ma-Jong | Never=0, seldom=1, every week=2, everyday=3 |
| Going to a club | No=0, yes=1 |
| Joining in an organization | No=0, yes=1 |
| Doing charity work | No=0, yes=1 |
| Attending a train course | No=0, yes=1 |
| Doing stock investment | No=0, yes=1 |
| Using the internet | No=0, yes=1 |
| Providing help | No=0, yes=1 |

**Supplementary Table 2. Sample characteristics of cognitive reserve markers**

| | Men (N=3,459) | Women (N=3,336) |
|---|---|---|
| **Childhood** | | |
| ***Starved at 0-5 years old*** (%) | | |
| Yes | 834 (24.1) | 829 (24.9) |
| No | 2045 (59.1) | 1976 (59.2) |
| Missing | 580 (16.8) | 531 (15.9) |
| ***Staved at 6-12 years old*** (%) | | |
| Yes | 1588 (45.9) | 1523 (45.7) |
| No | 1291 (37.3) | 1282 (38.4) |
| Missing | 580 (16.8) | 531 (15.9) |
| ***Starved at 13-17 years old*** (%) | | |
| Yes | 1475 (42.6) | 1408 (42.2) |
| No | 1404 (40.6) | 1397 (41.9) |
| Missing | 580 (16.8) | 531 (15.9) |
| ***Financial situation*** (%) | | |
| Worst | 740 (21.4) | 698 (20.9) |
| Worse | 445 (12.9) | 433 (13.0) |
| Average | 1467 (42.4) | 1386 (41.5) |
| Better | 193 (5.6) | 250 (7.5) |
| Best | 26 (0.8) | 41 (1.2) |
| Missing | 588 (17.0) | 528 (15.8) |
| ***Parents' education*** (%) | | |
| No formal education / illiterate | 2303 (66.6) | 2370 (71.0) |
| Capable of reading and/or writing | 346 (10.0) | 272 (8.2) |
| Sishu | 421 (12.2) | 324 (9.7) |
| Elementary school | 220 (6.4) | 207 (6.2) |
| Middle school | 66 (1.9) | 72 (2.2) |
| High school | 35 (1.0) | 23 (0.7) |
| Vocational school | 21 (0.6) | 25 (0.7) |
| Two/Three-year college/associate degree | 8 (0.2) | 6 (0.2) |
| Four-year college/bachelor's degree | 18 (0.5) | 13 (0.4) |
| Missing | 21 (0.6) | 24 (0.7) |
| ***Parents' occupation*** (%) | | |
| Agricultural | 2366 (68.4) | 2353 (70.5) |
| Non-agricultural | 477 (13.8) | 411 (12.3) |
| Missing | 616 (17.8) | 572 (17.1) |
| ***Height*** (m, mean, SD) | 1.62 (0.07) | 1.51 (0.06) |
| **Adulthood** | | |
| ***Education*** (%) | | |
| No formal education/illiterate | 632 (18.3) | 1763 (52.8) |
| Capable of reading and/or writing | 731 (21.1) | 601 (18.0) |

| | | |
|---|---|---|
| Sishu | 63 (1.8) | 11 (0.3) |
| Elementary school | 1095 (31.7) | 558 (16.7) |
| Middle school | 572 (16.5) | 268 (8.0) |
| High school | 97 (2.8) | 50 (1.5) |
| Vocational school | 160 (4.6) | 51 (1.5) |
| Two/Three-year college/associate degree | 62 (1.8) | 17 (0.5) |
| Four-year college/bachelor's degree | 45 (1.3) | 16 (0.5) |
| Post-graduate (master) | 2 (0.1) | 1 (0.0) |
| ***Highest occupational class*** (%) | | |
| Agricultural | 2118 (61.2) | 2376 (71.2) |
| Helper | 79 (2.3) | 104 (3.1) |
| Self-employment | 179 (5.2) | 117 (3.5) |
| Government or institution | 375 (10.8) | 158 (4.7) |
| Company/enterprise | 396 (11.4) | 307 (9.2) |
| Individual firm | 181 (5.2) | 67 (2.0) |
| Missing | 131 (3.8) | 207 (6.2) |
| **Later life** | | |
| ***Visiting friends*** (%) | | |
| Never | 2446 (70.7) | 2155 (64.6) |
| Seldom | 272 (7.9) | 283 (8.5) |
| Every week | 231 (6.7) | 238 (7.1) |
| Everyday | 499 (14.4) | 646 (19.4) |
| Missing | 11 (0.3) | 14 (0.4) |
| ***Playing Ma-Jong*** (%) | | |
| Never | 2750 (79.5) | 2957 (88.6) |
| Seldom | 211 (6.1) | 102 (3.1) |
| Every week | 240 (6.9) | 113 (3.4) |
| Everyday | 247 (7.1) | 150 (4.5) |
| Missing | 11 (0.3) | 14 (0.4) |
| ***Going to a club*** (%) | | |
| No | 3208 (92.7) | 3108 (93.2) |
| Yes | 240 (6.9) | 214 (6.4) |
| Missing | 11 (0.3) | 14 (0.4) |
| ***Joining an organization*** (%) | | |
| No | 3396 (98.2) | 3276 (98.2) |
| Yes | 52 (1.5) | 46 (1.4) |
| Missing | 11 (0.3) | 14 (0.4) |
| ***Doing charity work*** (%) | | |
| No | 3429 (99.1) | 3314 (99.3) |
| Yes | 19 (0.5) | 8 (0.2) |
| Missing | 11 (0.3) | 14 (0.4) |
| ***Attending a train course*** (%) | | |
| No | 3441 (99.5) | 3322 (99.6) |
| Yes | 7 (0.2) | 0 (0.0) |

| | | |
|---|---|---|
| Missing | 11 (0.3) | 14 (0.4) |
| ***Doing stock investment*** (%) | | |
| No | 3425 (99.0) | 3321 (99.6) |
| Yes | 23 (0.7) | 1 (0.0) |
| Missing | 11 (0.3) | 14 (0.4) |
| ***Using the internet*** (%) | | |
| No | 3395 (98.1) | 3300 (98.9) |
| Yes | 53 (1.5) | 22 (0.7) |
| Missing | 11 (0.3) | 14 (0.4) |
| ***Providing help to someone*** (%) | | |
| No | 3267 (94.4) | 3186 (95.5) |
| Yes | 181 (5.2) | 136 (4.1) |
| Missing | 11 (0.3) | 14 (0.4) |

*Note:* SD: standard deviation

**Supplementary Table 3. Unconditional latent growth curve models**

| | Mean (95% CI) | Variance (95% CI) |
|---|---|---|
| **Full sample (N=6,795)** | | |
| Intercept | 13.294 (13.147, 13.441) | 22.868 (21.577, 24.159) |
| Slope | -0.476 (-0.546, -0.407) | 0.108 (0.059, 0.157) |
| Quadratic slope | 0.014 (0.004, 0.025) | 0.000 (0.000,0.000) |
| **Men (N=3,459)** | | |
| Intercept | 14.721 (14.529, 14.912) | 19.627 (16.222, 23.032) |
| Slope | -0.459 (-0.558, -0.360) | 0.847 (-0.048, 1.743) |
| Quadratic slope | 0.015 (0.000, 0.029) | 0.009 (-0.005, 0.024) |
| **Women (N=3,336)** | | |
| Intercept | 11.782 (11.569, 11.996) | 23.605 (21.737, 25.472) |
| Slope | -0.485 (-0.586, -0.384) | 0.082 (0.012, 0.152) |
| Quadratic slope | 0.014 (-0.001, 0.029) | 0.000 (0.000, 0.000) |
| **Urban residence (N=2,701)** | | |
| Intercept | 15.010 (14.783, 15.237) | 24.392 (20.249, 28.535) |
| Slope | -0.394 (-0.508, -0.281) | 0.957 (-0.099, 2.013) |
| Quadratic slope | 0.012 (-0.005, 0.029) | 0.010 (-0.006, 0.027) |
| **Rural residence (N=4,094)** | | |
| Intercept | 12.144 (11.96, 12.329) | 19.863 (18.312, 21.414) |
| Slope | -0.498 (-0.588, -0.408) | 0.076 (0.014, 0.138) |
| Quadratic slope | 0.014 (0.001, 0.027) | 0.000 (0.000, 0.000) |
| **MCI subgroup (N=1,006)** | | |
| Intercept | 5.751 (5.539, 5.963) | 8.734 (7.196, 10.272) |
| Slope | 1.244 (1.076, 1.412) | 0.247 (0.139, 0.355) |
| Quadratic slope | -0.183 (-0.212, -0.154) | 0.000 (0.000,0.000) |
| **Cognitive normal subgroup (N=5,789)** | | |
| Intercept | 14.784 (14.645, 14.922) | 16.683 (15.754, 17.790) |
| Slope | -0.847 (-0.919, -0.775) | 0.203 (0.152, 0.255) |
| Quadratic slope | 0.053 (0.042, 0.063) | 0.000 (0.000,0.000) |

*Note:* CI: confidence interval

**Supplementary Table 4. Seven-year trajectories of global cognition (0-32) by cognitive reserve in men (N=3,459)**

| | Model 1* | | Model 2† | | Model 3‡ | |
|---|---|---|---|---|---|---|
| | Intercept | Slope | Intercept | Slope | Intercept | Slope |
| | b (95% CI) | b (95% CI) | b (95% CI) | b (95% CI) | b (95% CI) | b (95% CI) |
| **Mean** | 15.630 (15.250, 16.010) | -0.325 (-0.450, -0.200) | 15.391 (14.740, 16.041) | -0.411 (-0.591, -0.231) | 15.976 (15.323, 16.630) | -0.461 (-0.644, -0.279) |
| **Variance** | 7.979 (6.867, 9.091) | 0.107 (0.043, 0.172) | 7.778 (6.668, 8.887) | 0.100 (0.036, 0.164) | 7.159 (6.082, 8.236) | 0.097 (0.034, 0.161) |
| **Cognitive reserve** | 1.634 (1.538, 1.731) | 0.059 (0.035, 0.083) | 1.606 (1.509, 1.704) | 0.055 (0.031, 0.079) | 1.512 (1.414, 1.610) | 0.061 (0.036, 0.085) |
| **Baseline age, centred** | -0.196 (-0.264, -0.129) | -0.022 (-0.039, -0.006) | -0.188 (-0.256, -0.120) | -0.022 (-0.039, -0.005) | -0.178 (-0.245, -0.111) | -0.022 (-0.039, -0.005) |
| **Baseline age, squared** | 0.000 (-0.003, 0.004) | 0.000 (-0.001, 0.001) | 0.000 (-0.003, 0.004) | 0.000 (-0.001, 0.001) | 0.000 (-0.003, 0.003) | 0.000 (-0.001, 0.001) |
| **Urban-rural residence** | | | | | | |
| Urban | Ref | Ref | Ref | Ref | Ref | Ref |
| Rural | -0.594 (-0.914, -0.275) | 0.024 (-0.051, 0.099) | -0.471 (-0.796, -0.145) | 0.034 (-0.043, 0.110) | -0.337 (-0.658, -0.017) | 0.022 (-0.055, 0.099) |
| **Marital status** | | | | | | |
| Unmarried | | | Ref | Ref | Ref | Ref |
| Married | | | 0.187 (-0.262, 0.637) | 0.149 (0.037, 0.261) | 0.087 (-0.355, 0.529) | 0.148 (0.036, 0.260) |
| **Alcohol drinking** | | | | | | |
| Never | | | Ref | Ref | Ref | Ref |
| 1/month | | | -0.100 (-1.370, 1.171) | 0.156 (-0.162, 0.474) | 0.063 (-1.185, 1.310) | 0.138 (-0.180, 0.456) |
| 2-3/month | | | 0.157 (-0.715, 1.030) | -0.033 (-0.232, 0.167) | 0.162 (-0.693, 1.017) | -0.034 (-0.234, 0.165) |
| 1-6/week | | | 0.095 (-0.511, 0.701) | -0.012 (-0.154, 0.130) | 0.026 (-0.571, 0.622) | -0.013 (-0.155, 0.129) |
| ≥1/day | | | -0.169 (-0.544, 0.205) | 0.035 (-0.052, 0.123) | -0.308 (-0.677, 0.060) | 0.050 (-0.037, 0.138) |
| **Smoking status** | | | | | | |
| Never | | | Ref | Ref | Ref | Ref |
| Former | | | 0.223 (-0.203, 0.650) | -0.104 (-0.206, -0.003) | 0.377 (-0.042, 0.797) | -0.122 (-0.223, -0.020) |
| Current | | | -0.175 (-0.530, 0.179) | -0.098 (-0.180, -0.016) | -0.179 (-0.528, 0.169) | -0.097 (-0.178, -0.015) |
| **BMI** | | | | | | |

| | | | | |
|---|---|---|---|---|
| Underweight | -0.786 (-1.332, -0.239) | 0.078 (-0.059, 0.214) | -0.671 (-1.211, -0.131) | 0.075 (-0.062, 0.212) |
| Normal | Ref | Ref | Ref | Ref |
| Overweight | 0.345 (-0.053, 0.743) | 0.017 (-0.075, 0.109) | 0.304 (-0.093, 0.701) | 0.009 (-0.084, 0.102) |
| Obesity | 0.387 (-0.255, 1.030) | 0.039 (-0.110, 0.187) | 0.338 (-0.311, 0.988) | 0.012 (-0.141, 0.165) |
| **Hearing loss** | | | | |
| No | | | Ref | Ref |
| Yes | | | -0.912 (-1.341, -0.483) | 0.023 (-0.087, 0.133) |
| **Hypertension** | | | | |
| No | | | Ref | Ref |
| Yes | | | 0.070 (-0.261, 0.400) | 0.002 (-0.078, 0.081) |
| **Diabetes** | | | | |
| No | | | Ref | Ref |
| Yes | | | -0.074 (-0.656, 0.507) | -0.069 (-0.209, 0.072) |
| **Heart diseases** | | | | |
| No | | | Ref | Ref |
| Yes | | | -0.007 (-0.415, 0.401) | 0.006 (-0.091, 0.104) |
| **Stroke** | | | | |
| No | | | Ref | Ref |
| Yes | | | -0.144 (-0.882, 0.594) | -0.121 (-0.320, 0.077) |
| **Dyslipidaemia** | | | | |
| No | | | Ref | Ref |
| Yes | | | -0.072 (-0.586, 0.443) | 0.133 (0.012, 0.254) |
| **Probable depression**[§] | | | | |
| No | | | | |
| Yes | | | -1.089 (-1.446, -0.732) | 0.078 (-0.009, 0.164) |
| **ADLs (0-6)** | | | -0.497 (-0.653, -0.340) | 0.068 (0.025, 0.111) |

*Notes*: ADLs: activities of daily life; BMI: body mass index; Ref: reference category

Quadratic slope was not regressed on CR or covariates because the magnitude of the slope is too small (b=0.015)

* Adjusted for age, age squared, gender, and urban/rural residence. Model fit indices: χ2 = 58.075., $P < 0.001$; CFI = 0.991; TLI = 0.984; SRMR = 0.017; RMSEA = 0.033 (0.025, 0.042)

†Adjusted for Model 1 covariates plus smoking status, alcohol drinking frequency and BMI. Model fit indices: $\chi^2$=77.481, $P < 0.001$; CFI = 0.991; TLI = 0.983; SRMR = 0.009; RMSEA = 0.020 (0.014, 0.025)

‡Adjusted for Model 2 covariates plus number of limitations in ADLs, self-rated hearing, probable depression, and self-reported doctor-diagnosis of cardiovascular disease, hypertension, diabetes, stroke and dyslipidaemia. Model fit indices: $\chi^2$ = 91.933, $P < 0.001$; CFI = 0.992; TLI = 0.984; SRMR = 0.008; RMSEA = 0.016 (0.011, 0.021)

§Probable depression was defined as having CES-D-10 score≥12

**Supplementary Table 5. Seven-year trajectories of global cognition (0-32) by cognitive reserve in women (N=3,336)**

| | Model 1* | | Model 2† | | Model 3‡ | |
|---|---|---|---|---|---|---|
| | Intercept b (95% CI) | Slope b (95% CI) | Intercept b (95% CI) | Slope b (95% CI) | Intercept b (95% CI) | Slope b (95% CI) |
| **Mean** | 14.955 (14.592, 15.319) | -0.245 (-0.373, -0.118) | 14.345 (13.825, 14.865) | -0.217 (-0.371, -0.062) | 14.776 (14.240, 15.311) | -0.259 (-0.418, -0.101) |
| **Variance** | 8.999 (7.819, 10.179) | 8.999 (7.819, 10.179) | 8.809 (7.636, 9.981) | 0.08 (0.014, 0.146) | 8.335 (7.189, 9.481) | 0.075 (0.010, 0.141) |
| **Cognitive reserve** | 1.961 (1.860, 2.062) | 0.068 (0.045, 0.091) | 1.946 (1.844, 2.047) | 0.066 (0.043, 0.089) | 1.861 (1.759, 1.963) | 0.071 (0.047, 0.095) |
| **Baseline age, centred** | -0.206 (-0.272, -0.141) | -0.025 (-0.043, -0.008) | -0.196 (-0.262, -0.130) | -0.025 (-0.042, -0.008) | -0.188 (-0.253, -0.122) | -0.026 (-0.043, -0.009) |
| **Baseline age, squared** | 0.001 (-0.002, 0.003) | 0.001 (0.000, 0.001) | 0.001 (-0.002, 0.003) | 0.001 (0.000, 0.001) | 0.001 (-0.002, 0.003) | 0.001 (0.000, 0.001) |
| **Urban-rural residence** | | | | | | |
| Urban | Ref | Ref | Ref | Ref | Ref | Ref |
| Rural | -0.868 (-1.206, -0.530) | -0.071 (-0.150, 0.007) | -0.837 (-1.179, -0.496) | -0.061 (-0.141, 0.019) | -0.647 (-0.987, -0.306) | -0.067 (-0.148, 0.013) |
| **Marital status** | | | | | | |
| Unmarried | | | Ref | Ref | Ref | Ref |
| Married | | | 0.259 (-0.106, 0.623) | -0.002 (-0.090, 0.085) | 0.189 (-0.172, 0.549) | 0.003 (-0.084, 0.090) |
| **Alcohol drinking** | | | | | | |
| Never | | | Ref | Ref | Ref | Ref |
| 1/month | | | -0.219 (-2.583, 2.146) | 0.106 (-0.381, 0.593) | -0.218 (-2.555, 2.118) | 0.122 (-0.364, 0.607) |
| 2-3/month | | | -0.884 (-2.530, 0.762) | 0.278 (-0.097, 0.652) | -0.832 (-2.455, 0.792) | 0.264 (-0.109, 0.637) |
| 1-6/week | | | 0.512 (-0.967, 1.990) | -0.205 (-0.533, 0.122) | 0.619 (-0.841, 2.080) | -0.220 (-0.547, 0.107) |
| ≥1/day | | | 0.878 (-0.267, 2.024) | -0.217 (-0.490, 0.057) | 1.019 (-0.113, 2.152) | -0.224 (-0.496, 0.049) |
| **Smoking status** | | | | | | |
| Never | | | Ref | Ref | Ref | Ref |
| Former | | | 0.141 (-0.744, 1.025) | 0.016 (-0.199, 0.231) | 0.289 (-0.585, 1.163) | 0.000 (-0.214, 0.215) |
| Current | | | 0.389 (-0.184, 0.962) | -0.156 (-0.289, -0.023) | 0.479 (-0.087, 1.044) | -0.168 (-0.301, -0.035) |
| **BMI** | | | | | | |

| | | | | |
|---|---|---|---|---|
| Underweight | -0.026 (-0.627, 0.575) | -0.139 (-0.283, 0.005) | 0.046 (-0.549, 0.641) | -0.148 (-0.292, -0.004) |
| Normal | Ref | Ref | Ref | Ref |
| Overweight | 0.834 (0.443, 1.224) | -0.025 (-0.114, 0.063) | 0.735 (0.345, 1.125) | -0.011 (-0.100, 0.079) |
| Obesity | 0.146 (-0.385, 0.676) | -0.018 (-0.138, 0.102) | 0.065 (-0.477, 0.606) | 0.003 (-0.120, 0.127) |
| **Hearing loss** | | | | |
| No | | | Ref | Ref |
| Yes | | | -0.562 (-1.044, -0.080) | 0.088 (-0.035, 0.211) |
| **Hypertension** | | | | |
| No | | | Ref | Ref |
| Yes | | | -0.101 (-0.436, 0.234) | -0.018 (-0.097, 0.060) |
| **Diabetes** | | | | |
| No | | | Ref | Ref |
| Yes | | | 0.287 (-0.260, 0.834) | -0.070 (-0.199, 0.059) |
| **Heart diseases** | | | | |
| No | | | Ref | Ref |
| Yes | | | 0.390 (-0.017, 0.797) | 0.070 (-0.026, 0.167) |
| **Stroke** | | | | |
| No | | | Ref | Ref |
| Yes | | | -0.519 (-1.355, 0.317) | -0.115 (-0.322, 0.092) |
| **Dyslipidaemia** | | | | |
| No | | | Ref | Ref |
| Yes | | | 0.216 (-0.276, 0.709) | -0.021 (-0.134, 0.091) |
| **Probable depression**[§] | | | | |
| No | | | | |
| Yes | | | -1.008 (-1.345, -0.672) | 0.078 (-0.002, 0.157) |
| **ADLs (0-6)** | | | -0.350 (-0.490, -0.211) | 0.024 (-0.012, 0.060) |

*Notes*: ADLs: activities of daily life; BMI: body mass index; Ref: reference category

Quadratic slope was not regressed on CR or covariates because the magnitude of the slope is too small (b=0.014)

*Adjusted for age, age squared, gender, and urban/rural residence. Model fit indices: $\chi 2$ = 27.186, P < 0.05; CFI = 0.998; TLI = 0.996; SRMR = 0.010; RMSEA = 0.019 (0.010, 0.029)

†Adjusted for Model 1 covariates plus smoking status, alcohol drinking frequency and BMI. Model fit indices: $\chi^2$=44.008, P = 0.0768; CFI = 0.999; TLI = 0.996; SRMR = 0.006; RMSEA = 0.011 (0.000, 0.018)

‡Adjusted for Model 2 covariates plus number of limitations in ADLs, self-rated hearing, probable depression, and self-reported doctor-diagnosis of cardiovascular disease, hypertension, diabetes, stroke and dyslipidaemia. Model fit indices: $\chi^2$ = 54.401, P = 0.2439; CFI = 0.999; TLI = 0.998; SRMR = 0.005; RMSEA = 0.006 (0.000, 0.013)

§Probable depression was defined as having CES-D-10 score≥12

**Supplementary Table 6. Seven-year trajectories of global cognition (0-32) by cognitive reserve in adults with mild cognitive impairments (N=1,006)**

| | Model 1* | | Model 2† | | Model 3‡ | |
|---|---|---|---|---|---|---|
| | Intercept | Slope | Intercept | Slope | Intercept | Slope |
| | b (95% CI) | b (95% CI) | b (95% CI) | b (95% CI) | b (95% CI) | b (95% CI) |
| **Mean** | 8.360 (7.955, 8.765) | 1.640 (1.396, 1.884) | 7.585 (6.963, 8.207) | 1.702 (1.380, 2.024) | 7.779 (7.148, 8.409) | 1.686 (1.361, 2.012) |
| **Variance** | 2.673 (1.658, 3.687) | 0.219 (0.129, 0.310) | 2.532 (1.531, 3.533) | 0.205 (0.116, 0.294) | 2.404 (1.410, 3.399) | 0.195 (0.107, 0.284) |
| **Cognitive reserve** | 1.319 (1.226, 1.412) | 0.124 (0.084, 0.164) | 1.313 (1.220, 1.406) | 0.123 (0.083, 0.163) | 1.298 (1.204, 1.391) | 0.125 (0.085, 0.166) |
| **Baseline age, centred** | -0.190 (-0.245, -0.136) | -0.042 (-0.073, -0.011) | -0.178 (-0.233, -0.123) | -0.039 (-0.069, -0.008) | -0.175 (-0.230, -0.121) | -0.039 (-0.070, -0.008) |
| **Baseline age, squared** | 0.001 (-0.001, 0.003) | 0.001 (0.000, 0.003) | 0.001 (-0.002, 0.003) | 0.001 (0.000, 0.003) | 0.001 (-0.001, 0.003) | 0.001 (-0.001, 0.003) |
| **Gender** | | | | | | |
| Men | Ref | Ref | Ref | Ref | Ref | Ref |
| Women | -0.042 (-0.355, 0.270) | -0.105 (-0.243, 0.032) | 0.372 (-0.050, 0.794) | -0.162 (-0.354, 0.029) | 0.390 (-0.031, 0.810) | -0.158 (-0.350, 0.033) |
| **Urban-rural residence** | | | | | | |
| Urban | Ref | Ref | Ref | Ref | Ref | Ref |
| Rural | -0.247 (-0.565, 0.071) | -0.055 (-0.198, 0.089) | -0.240 (-0.556, 0.075) | -0.050 (-0.192, 0.093) | -0.220 (-0.537, 0.096) | -0.056 (-0.199, 0.088) |
| **Marital status** | | | | | | |
| Unmarried | | | Ref | Ref | Ref | Ref |
| Married | | | 0.351 (0.013, 0.688) | -0.027 (-0.182, 0.128) | 0.325 (-0.013, 0.662) | -0.021 (-0.176, 0.134) |
| **Alcohol drinking** | | | | | | |
| Never | | | Ref | Ref | Ref | Ref |
| 1/month | | | 0.433 (-1.485, 2.352) | 0.001 (-0.675, 0.676) | 0.448 (-1.465, 2.360) | 0.001 (-0.674, 0.677) |
| 2-3/month | | | -0.241 (-1.219, 0.737) | 0.595 (0.187, 1.002) | -0.316 (-1.291, 0.659) | 0.616 (0.209, 1.023) |
| 1-6/week | | | 0.897 (0.032, 1.763) | -0.008 (-0.401, 0.385) | 0.848 (-0.014, 1.710) | -0.016 (-0.411, 0.379) |
| ≥1/day | | | 0.573 (0.060, 1.085) | 0.033 (-0.182, 0.248) | 0.565 (0.055, 1.076) | 0.035 (-0.181, 0.252) |
| **Smoking status** | | | | | | |
| Never | | | Ref | Ref | Ref | Ref |

| | | | | |
|---|---|---|---|---|
| Former | 0.698 (0.134, 1.262) | -0.141 (-0.398, 0.117) | 0.737 (0.171, 1.303) | -0.134 (-0.393, 0.124) |
| Current | 0.104 (-0.316, 0.524) | -0.113 (-0.295, 0.070) | 0.085 (-0.334, 0.504) | -0.114 (-0.296, 0.069) |
| **BMI** | | | | |
| Underweight | -0.055 (-0.539, 0.428) | -0.136 (-0.358, 0.087) | -0.001 (-0.484, 0.482) | -0.157 (-0.379, 0.065) |
| Normal | Ref | Ref | Ref | Ref |
| Overweight | 0.281 (-0.097, 0.659) | 0.025 (-0.131, 0.181) | 0.238 (-0.145, 0.621) | 0.024 (-0.135, 0.183) |
| Obesity | -0.451 (-0.969, 0.067) | 0.068 (-0.153, 0.288) | -0.546 (-1.087, -0.006) | 0.085 (-0.148, 0.318) |
| **Hearing loss** | | | | |
| No | | | Ref | Ref |
| Yes | | | -0.137 (-0.497, 0.223) | 0.079 (-0.086, 0.243) |
| **Hypertension** | | | | |
| No | | | Ref | Ref |
| Yes | | | 0.057 (-0.248, 0.362) | -0.095 (-0.231, 0.040) |
| **Diabetes** | | | | |
| No | | | Ref | Ref |
| Yes | | | -0.323 (-0.888, 0.241) | 0.041 (-0.205, 0.286) |
| **Heart diseases** | | | | |
| No | | | Ref | Ref |
| Yes | | | 0.078 (-0.305, 0.461) | 0.067 (-0.104, 0.238) |
| **Stroke** | | | | |
| No | | | Ref | Ref |
| Yes | | | 0.129 (-0.524, 0.782) | -0.436 (-0.788, -0.084) |
| **Dyslipidaemia** | | | | |
| No | | | Ref | Ref |
| Yes | | | 0.171 (-0.328, 0.670) | -0.035 (-0.249, 0.178) |
| **Probable depression**§ | | | | |

| | | | |
|---|---|---|---|
| No | | | |
| Yes | | -0.330 (-0.624, -0.037) | 0.057 (-0.071, 0.186) |
| **ADLs (0-6)** | | -0.107 (-0.213, -0.001) | 0.024 (-0.029, 0.076) |

*Notes*: ADLs: activities of daily life; BMI: body mass index; Ref: reference category

Quadratic slope was not regressed on CR or covariates because the magnitude of the slope is too small (b=-0.183)

Mild cognitive impairments were determined by overall cognition scores which are lower than 1 standard deviation below the mean for their age and education matched peers

*Adjusted for age, age squared, gender, and urban/rural residence. Model fit indices: $\chi^2$ = 125.489, P < 0.001; CFI = 0.940; TLI = 0.889; SRMR = 0.043; RMSEA = 0.089 (0.075, 0.104)

†Adjusted for Model 1 covariates plus smoking status, alcohol drinking frequency and BMI. Model fit indices: χ2 = 146.579, P < 0.001; CFI = 0.940; TLI=0.884; SRMR = 0.025; RMSEA = 0.057 (0.048, 0.067)

‡Adjusted for Model 2 covariates plus number of limitations in ADLs, self-rated hearing, probable depression, and self-reported doctor-diagnosis of cardiovascular disease, hypertension, diabetes, stroke and dyslipidaemia. Model fit indices: χ2 = 161.900, P < 0.001; CFI = 0.941; TLI = 0.884; SRMR = 0.020; RMSEA = 0.047 (0.039, 0.055)

§Probable depression was defined as having CES-D-10 score≥12

**Supplementary Table 7. Seven-year trajectories of global cognition (0-32) by cognitive reserve in adults with normal cognition (N=5,789)**

| | Model 1* | | Model 2† | | Model 3‡ | |
|---|---|---|---|---|---|---|
| | Intercept b (95% CI) | Slope b (95% CI) | Intercept b (95% CI) | Slope b (95% CI) | Intercept b (95% CI) | Slope b (95% CI) |
| **Mean** | 16.682 (16.406, 16.958) | -0.667 (-0.764, -0.569) | 16.462 (16.018, 16.907) | -0.686 (-0.822, -0.551) | 16.799 (16.351, 17.247) | -0.709 (-0.846, -0.572) |
| **Variance** | 5.586 (4.877, 6.296) | 0.173 (0.126, 0.221) | 5.497 (4.789, 6.206) | 0.171 (0.124, 0.218) | 5.197 (4.501, 5.893) | 0.169 (0.122, 0.216) |
| **Cognitive reserve** | 1.554 (1.488, 1.621) | 0.097 (0.079, 0.115) | 1.537 (1.470, 1.604) | 0.094 (0.076, 0.112) | 1.467 (1.399, 1.535) | 0.095 (0.077, 0.114) |
| **Baseline age, centred** | -0.194 (-0.240, -0.149) | -0.025 (-0.037, -0.012) | -0.187 (-0.233, -0.141) | -0.024 (-0.036, -0.011) | -0.182 (-0.228, -0.137) | -0.024 (-0.037, -0.011) |
| **Baseline age, squared** | -0.001 (-0.003, 0.001) | 0.000 (0.000, 0.001) | -0.001 (-0.003, 0.002) | 0.000 (0.000, 0.001) | -0.001 (-0.003, 0.002) | 0.000 (0.000, 0.001) |
| **Gender** | | | | | | |
| Men | Ref | Ref | Ref | Ref | Ref | Ref |
| Women | -0.581 (-0.790, -0.371) | -0.002 (-0.057, 0.053) | -0.702 (-0.974, -0.429) | -0.038 (-0.110, 0.033) | -0.618 (-0.889, -0.346) | -0.046 (-0.117, 0.026) |
| **Urban-rural residence** | | | | | | |
| Urban | Ref | Ref | Ref | Ref | Ref | Ref |
| Rural | -0.519 (-0.735, -0.302) | -0.046 (-0.103, 0.011) | -0.433 (-0.653, -0.213) | -0.041 (-0.099, 0.018) | -0.301 (-0.521, -0.082) | -0.043 (-0.102, 0.015) |
| **Marital status** | | | | | | |
| Unmarried | | | Ref | Ref | Ref | Ref |
| Married | | | 0.207 (-0.062, 0.475) | 0.060 (-0.014, 0.134) | 0.133 (-0.133, 0.398) | 0.063 (-0.011, 0.136) |
| **Alcohol drinking** | | | | | | |
| Never | | | Ref | Ref | Ref | Ref |
| 1/month | | | -0.555 (-1.567, 0.457) | 0.209 (-0.069, 0.487) | -0.423 (-1.424, 0.579) | 0.209 (-0.069, 0.487) |
| 2-3/month | | | 0.099 (-0.630, 0.828) | -0.057 (-0.243, 0.128) | 0.149 (-0.572, 0.869) | -0.059 (-0.245, 0.127) |
| 1-6/week | | | -0.031 (-0.545, 0.484) | -0.027 (-0.159, 0.106) | -0.057 (-0.565, 0.452) | -0.026 (-0.159, 0.106) |
| ≥1/day | | | -0.191 (-0.519, 0.137) | 0.020 (-0.065, 0.106) | -0.268 (-0.592, 0.057) | 0.028 (-0.058, 0.113) |
| **Smoking status** | | | | | | |
| Never | | | Ref | Ref | Ref | Ref |

| | | | | |
|---|---|---|---|---|
| Former | 0.078 (-0.265, 0.422) | -0.067 (-0.159, 0.025) | 0.173 (-0.167, 0.514) | -0.072 (-0.165, 0.020) |
| Current | -0.153 (-0.428, 0.122) | -0.072 (-0.144, -0.001) | -0.124 (-0.396, 0.148) | -0.073 (-0.144, -0.002) |
| **BMI** | | | | |
| Underweight | -0.491 (-0.878, -0.104) | -0.026 (-0.132, 0.081) | -0.423 (-0.807, -0.038) | -0.026 (-0.133, 0.081) |
| Normal | Ref | Ref | Ref | Ref |
| Overweight | 0.346 (0.086, 0.607) | 0.024 (-0.044, 0.092) | 0.289 (0.028, 0.550) | 0.023 (-0.046, 0.092) |
| Obesity | 0.224 (-0.160, 0.608) | 0.018 (-0.082, 0.119) | 0.152 (-0.239, 0.543) | 0.012 (-0.091, 0.116) |
| **Hearing loss** | | | | |
| No | | | Ref | Ref |
| Yes | | | -0.504 (-0.819, -0.189) | 0.000 (-0.090, 0.090) |
| **Hypertension** | | | | |
| No | | | Ref | Ref |
| Yes | | | -0.072 (-0.295, 0.151) | 0.002 (-0.058, 0.061) |
| **Diabetes** | | | | |
| No | | | Ref | Ref |
| Yes | | | 0.079 (-0.293, 0.452) | -0.071 (-0.172, 0.030) |
| **Heart diseases** | | | | |
| No | | | Ref | Ref |
| Yes | | | 0.24 (-0.031, 0.511) | 0.040 (-0.033, 0.113) |
| **Stroke** | | | | |
| No | | | Ref | Ref |
| Yes | | | -0.13 (-0.663, 0.403) | -0.089 (-0.242, 0.064) |
| **Dyslipidaemia** | | | | |
| No | | | Ref | Ref |
| Yes | | | 0.183 (-0.147, 0.514) | 0.064 (-0.024, 0.151) |
| **Probable depression**[§] | | | | |

| | | | | |
|---|---|---|---|---|
| No | | | | |
| Yes | | | -0.896 (-1.132, -0.660) | 0.046 (-0.018, 0.110) |
| **ADLs (0-6)** | | | -0.300d (-0.406, -0.194) | 0.021 (-0.010, 0.051) |

*Notes*: ADLs: activities of daily life; BMI: body mass index; Ref: reference category

Quadratic slope was not regressed on CR or covariates because the magnitude of the slope is too small (b=0.053)

Normal cognition was determined by overall cognition scores which are higher than 1 standard deviation below the mean for their age and education matched peers

*Adjusted for age, age squared, gender, and urban/rural residence. Model fit indices: $\chi 2$ = 94.919, P < 0.001; CFI = 0.992; TLI = 0.985; SRMR = 0.024; RMSEA = 0.032 (0.026, 0.038)

†Adjusted for Model 1 covariates plus smoking status, alcohol drinking frequency and BMI. Model fit indices: $\chi 2$ = 112.574, P < 0.001; CFI = 0.992; TLI = 0.985; SRMR = 0.013; RMSEA = 0.020 (0.016, 0.024)

‡Adjusted for Model 2 covariates plus number of limitations in ADLs, self-rated hearing, probable depression, and self-reported doctor-diagnosis of cardiovascular disease, hypertension, diabetes, stroke and dyslipidaemia. Model fit indices: $\chi 2$ = 124.104, P < 0.001; CFI = 0.993; TLI = 0.986; SRMR = 0.009; RMSEA = 0.016 (0.012, 0.019)

§ Probable depression was defined as having CES-D-10 score$\geq$1
